# Lytic cycle of *Besnoitia besnoiti* tachyzoites displays similar features in primary bovine endothelial cells and fibroblasts

**DOI:** 10.1186/s13071-019-3777-0

**Published:** 2019-11-04

**Authors:** Alejandro Jiménez-Meléndez, María Fernández-Álvarez, Alexandra Calle, Miguel Ángel Ramírez, Carlos Diezma-Díaz, Patricia Vázquez-Arbaizar, Luis Miguel Ortega-Mora, Gema Álvarez-García

**Affiliations:** 1 SALUVET, Animal Health Department, Faculty of Veterinary Sciences, Complutense University of Madrid, Ciudad Universitaria s/n, 28040 Madrid, Spain; 2 Departamento de Reproducción Animal, Instituto Nacional de Investigación y Tecnología Agraria y Alimentaria (INIA), Avenida Puerta de Hierro 12, local 10, 28040 Madrid, Spain

**Keywords:** *Besnoitia besnoiti*, Lytic cycle, Primary aorta endothelial cells, Fibroblasts, Flow cytometry, Bovine viral diarrhoea virus

## Abstract

**Background:**

Bovine besnoitiosis, caused by the cyst-forming apicomplexan parasite *Besnoitia besnoiti*, is a chronic and debilitating cattle disease that continues to spread in Europe in the absence of control tools. In this scenario, *in vitro* culture systems are valuable tools to carry out drug screenings and to unravel host-parasite interactions. However, studies performed in bovine target cells are scarce.

**Methods:**

The objective of the present study was to obtain primary bovine aortic endothelial cells (BAECs) and fibroblast cell cultures, target cells during the acute and the chronic stage of the disease, respectively, from healthy bovine donors. Afterwards, expression of surface (CD31, CD34 and CD44) and intracellular markers (vimentin and cytokeratin) was studied to characterize cell populations by flow cytometry. Next, the lytic cycle of *B. besnoiti* tachyzoites was studied in both target cells. Invasion rates (IRs) were determined by immunofluorescence at several time points post-infection, and proliferation kinetics were studied by quantitative PCR (qPCR). Finally, the influence of bovine viral diarrhea virus (BVDV) co-infection on the host cell machinery, and consequently on *B. besnoiti* invasion and proliferation, was investigated in BAECs.

**Results:**

Morphology and cytometry results confirmed the endothelial and fibroblast origins. CD31 was the surface marker that best discriminated between BAECs and fibroblasts, since fibroblasts lacked CD31 labelling. Expression of CD34 was weak in low-passage BAECs and absent in high-passage BAECs and fibroblasts. Positive labelling for CD44, vimentin and cytokeratin was observed in both BAECs and fibroblasts. Regarding the lytic cycle of the parasite, although low invasion rates (approximately 3–4%) were found in both cell culture systems, more invasion was observed in BAECs at 24 and 72 hpi. The proliferation kinetics did not differ between BAECs and fibroblasts. BVDV infection favoured early *Besnoitia* invasion but there was no difference in tachyzoite yields observed in BVDV-BAECs compared to BAECs.

**Conclusions:**

We have generated and characterized two novel standardized *in vitro* models for *Besnoitia besnoiti* infection based on bovine primary target BAECs and fibroblasts, and have shown the relevance of BVDV coinfections, which should be considered in further studies with other cattle pathogens.

## Background

Bovine besnoitiosis, caused by the cyst-forming apicomplexan parasite *Besnoitia besnoiti,* is a chronic and debilitating cattle disease characterized by both cutaneous and systemic clinical manifestations. This parasitic disease progresses in two sequential phases as a consequence of the development of the two asexual and infective stages of the parasite: tachyzoites, responsible for the acute infection, and bradyzoites, responsible for the chronic infection [1]. Acutely infected animals may develop fever, oedema, orchitis and respiratory disorders. It has been postulated that endothelial and mononuclear cells are the parasite target cells during this stage. The tachyzoite lytic cycle results in host cell invasion, proliferation and egress from infected cells with subsequent tissue damage that may result in degenerative and fibroid necrotic lesions, vasculitis and thrombosis in parasitized tissues [2–4]. Next, tachyzoites switch into bradyzoites, which are packed inside tissue cysts to evade host immune responses. Tissue cysts are responsible for the characteristic skin lesions, such as hyperkeratosis, folding, alopecia and scars that occur during the chronic stage [1]. Previous studies have shown that tissue cyst formation occurs predominantly in cells of mesenchymal origin, such as fibroblasts and myofibroblasts [5].

Currently, this parasitic disease continues to spread in Europe in the absence of control tools [6]. In this scenario, *in vitro* culture systems are essential tools to carry out safety and efficacy drug screenings and to unravel host-parasite interactions [7].

Tachyzoites of *B. besnoiti* can be successfully maintained in primary cultures and in immortalized cell lines from different host origins (tick, mouse, monkey, cat, hamster or human) [8–11]. However, it has been reported that primary cells maintain many of the important markers and functions seen *in vivo* and better mimic the *in vivo* environment [12]. Moreover, the host species seems to be crucial when dissecting host-pathogen interactions [7]. Studies performed so far with *B. besnoiti* in primary bovine cell lines have been restricted to the embryonic calf heart cells KH-R [10], bovine umbilical vein endothelial cells (BUVECs) [13, 14], as well as bovine monocytes and neutrophils [15, 16]. Nonetheless, BUVECs are unlikely to be infected in natural infections since vertical transmission has not been reported, and endothelial cell (ECs) populations show heterogeneity in structure and function, depending on their localization [17]. Thus, primary target ECs of the adult cattle circulatory system might be an appropriate tool. On the other hand, although *B. besnoiti* tachyzoites have been successfully maintained in human foreskin fibroblasts [11], these cells are of a different host origin and thus are not the ideal system to dissect host-parasite interactions at the molecular level.

Another key issue to be considered regarding *in vitro* systems is the avoidance of cell culture contaminants, such as *Mycoplasma* spp. and viral infections to obtain reproducible and reliable data [18]. There are commercially available bovine ECs and fibroblasts, both as established and as primary cultures. However, the presence of bovine pathogens, such as bovine viral diarrhoea virus, a prevalent bovine pathogen and frequent contaminant in foetal bovine serum batches worldwide [19], is not routinely checked. In addition, established cell lines in repositories have been confirmed to be infected with BVDV [18]. Since BVDV is known to alter the transcriptomic profile of ECs *in vitro* [20], it may also influence the interaction of *B. besnoiti* with these target host cells.

The objective of the present study was to obtain and characterize primary bovine endothelial and fibroblast cell cultures from healthy bovine donors. Next, the *B. besnoiti* lytic cycle was studied in both target cells in terms of invasion and proliferation capabilities. Finally, the influence of BVDV co-infection was investigated in ECs.

## Methods

### Donor animals

Heifers from the Asturiana de la Montaña breed that were approximately 24-months-old were selected based on the absence of pathogens that are commonly found in cattle, such as BVDV, infectious bovine rhinotracheitis, *Neospora caninum* and *Mycobacterium avium paratuberculosis* based on ELISA techniques. The presence of specific antibodies against *B. besnoiti* and *N. caninum* was also ruled out by Western blotting [21, 22].

### Isolation of primary endothelial cells and fibroblasts from bovine aortas

The selected animals were culled at a local slaughterhouse, and fresh bovine aortas were obtained and immediately clamped with sterilized plastic tie wraps. Next, the lumen was filled with wash medium consisting of Dulbeccoʼs modified Eagleʼs medium (DMEM high glucose, Gibco, Life Technologies, Thermo Fisher Scientific, Waltham, MA, USA), 10% FBS (Gibco) and a mixture of antibiotics (200 U/ml penicillin, 200 mg/ml streptomycin) (Lonza, Basel, Switzerland) [23]. Afterwards, under a sterile hood, the aortas were dipped in ethanol and were cut into two or three 5-cm long pieces, and each piece was opened with a longitudinal incision. The specimens were placed in separate sterile Petri dishes with the endothelium facing up and covered with sterile filter paper to avoid further digestion of the tissue [23]. Approximately 2 ml of 0.1% (wt/vol) collagenase type II solution was dripped onto each filter paper. Collagenase type II solution was prepared by dissolving 0.1 g of collagenase II (Gibco) in 100 ml of Hank’s balanced salt solution with calcium chloride (CaCl_2_) and magnesium chloride (MgCl_2_) (Gibco). Samples were incubated at 37 °C for 30 min, and the resulting fluid from the enzymatic digestion was centrifuged at 250× *g* at 4 °C for 5 min. The sample was then resuspended in specific medium for ECs, namely, M200 (Gibco), plus 20% FBS and a specific supplement for large vessel ECs (LVES, Gibco) prior to seeding the cells into T25 flasks. The medium was changed every 2–3 days until the cells were confluent and passaged.

For the isolation of ECs, differential trypsinization was performed to prevent fibroblast contamination according to previously published protocols [24]. Fibroblasts detach first in a mixed-cell culture when they are trypsinized. Briefly, when fibroblast contamination was detected, the cell culture medium was discarded, and the monolayer was gently rinsed with phosphate-buffered saline (PBS). Prewarmed trypsin-EDTA was added, and the area of the culture containing both bovine aorta ECs and fibroblasts was observed under a light inverted microscope (Nikon Eclipse TS100) until the first fibroblasts started to detach. The cell culture supernatant containing fibroblasts was discarded, and fresh culture medium was added to the flask with BAECs.

To isolate fibroblasts, in parallel, one flask was left undisturbed, without differential trypsinization, and fibroblasts slowly outgrew the ECs until the monolayer consisted only of fibroblasts. Afterwards, the fibroblasts were passaged, and a homogeneous culture was observed under the light microscope. Three trials and two aortas per trial were needed to succeed in the isolation of BAEC and fibroblasts. Once isolated, the same cells were used for all the experiments.

### Maintenance of *B. besnoiti* tachyzoites and cell cultures

Tachyzoites from the *B. besnoiti* Spain1 isolate (BbSpain1) were routinely maintained and propagated in the monkey kidney cell line MARC-145, according to previously published procedures [11]. MARC-145 cell cultures were passaged twice a week.

For *in vitro* assays, tachyzoites were harvested at three days post-infection, when most of the tachyzoites were still intracellular, by removing the infected cell monolayer with a cell scraper, followed by repeated passages through a 25-gauge needle and separation from cell debris on a disposable PD-10 column [11]. To avoid parasite adaptation to cell culture, only low-passage tachyzoites were included in the studies (passage numbers 10 to 21). Tachyzoite viability was confirmed by trypan blue exclusion followed by counting in a Neubauer chamber. Purified viable tachyzoites were used to infect confluent fibroblast or BAECs monolayers as described below for the lytic cycle characterization.

The *B. besnoiti* isolate used for all *in vitro* assays tested negative for *Mycoplasma* spp. infection by PCR (Mycoplasma Gel Form Kit®, Biotools, Madrid, Spain) following the manufacturerʼs instructions, and BVDV by quantitative real-time reverse-transcriptase PCR (RT-PCR) [25]. The FCS used in all experiments was previously tested to confirm the absence of IgGs against *B. besnoiti*, *N. caninum* and *Toxoplasma gondii* by IFAT [26], and BVDV by RT-PCR [25].

Newly obtained BAECs were maintained in a specific medium for ECs M200 (Gibco) with 20% FBS, LVES (Gibco) and a mixture of antibiotics (penicillin + streptomycin, Lonza, Basel, Switzerland) and passaged when confluent, approximately every 3–4 days. Bovine fibroblasts were cultured in DMEM with 15% FBS and antibiotics and passaged once a week using pre-mixed Trypsin EDTA (TrypLE®, Gibco). High-passage BAECs were maintained as described for low-passage BAECs. To avoid changes associated with the long-term *in vitro* maintenance of the cells, only low-passage BAECs and fibroblasts were used for the lytic cycle characterization.

Bovine pulmonary artery endothelial cells (CPAE, ATCC® CCL-209), that were persistently infected with a non-cytopathic strain of BVDV were maintained in Dulbeccoʼs modified Eagleʼs medium (DMEM) high glucose (Gibco) plus 15% FBS and antibiotics (100 U/ml penicillin, 100 mg/ml streptomycin) and passaged twice a week. To avoid cross-contamination, this cell line was kept in a different cell culture room with a separate incubator and laminar flow hood. This cell line was used as a source of infectious BVDV for further experiments.

Bovine immortalized endometrial cells (eMSC 4H) were employed as positive controls for the expression of the surface and intracellular markers that were analysed by flow cytometry. This cell line represents a cell population with markers that are characteristic of mesenchymal and epithelial cells. The eMSC 4H cells were maintained in RPMI 1640 with (Lonza, Basel, Switzerland), 10% FCS and antibiotics (100 U/ml penicillin, 100 mg/ml streptomycin), and were passaged once a week.

### Flow cytometry of surface and intracellular markers in bovine cell lines

The pattern of the expression of surface proteins (CD31, CD34, CD44) and intracellular molecules (vimentin and cytokeratin) was analysed by flow cytometry both on low- and high-passage BAECs (passages 8 and 28, respectively), low-passage fibroblasts (passage 10) and endometrial cells, essentially as previously described [27].

The immunocytochemical analysis by flow cytometry was carried out following procedures previously described [27]. Briefly, for intracellular markers, first, cells were fixed with paraformaldehyde and then permeabilized with 0.4% Triton X100. Afterwards, appropriate dilutions, which were recommended by the manufacturers, of the primary antibodies listed in Table 1 were added. Alexa Fluor 488-conjugated secondary antibodies (Jackson ImmunoResearch Laboratories, Cambridgeshire, UK) were added at a 1:500 dilution and incubated for 30 min at room temperature (RT). For CD31, which was conjugated with allophycocyanin (APC), cells were washed with PBS, and the appropriate dilution of the conjugated primary antibody (1:50) in TNB was added to the cells. Subsequently, the cells were washed twice with PBS. Cells that were incubated with only the appropriate secondary antibodies were used as negative control samples. The analysis of the samples to determine the fold change of the expression, which was based on the fluorescence relative to that of the negative control cells, was performed with the Cell Lab Quanta SC system of Beckman Coulter using the FlowJo X software version 10.0.7r2 (Beckton Dickinson, Franklin lakes, NJ, USA).

**Table 1:** Markers employed to characterize primary BAEC and bovine fibroblasts by flow cytometry

Marker	Location	Primary antibody			Secondary antibody		Labelled cell^a^	References
		Clone	Isotype	Dilution	Isotype (Alexa fluor 488)	Dilution		
Vimentin	Intracellular	LN-6^d^	Mouse monoclonal IgM	1:100	Anti Mouse IgG	1:500	ECs/Fb	[39, 42]
Cytokeratin	Intracellular	C-11^d^	Mouse monoclonal IgG1	1:100	Anti Mouse IgG	1:500	ECs/Fb	[43–45]
CD34^b^**	Surface	NA^e^	Rabbit polyclonal IgG	1:100	Anti Rabbit IgG	1:500	ECs	[35]
CD44	Surface	IM7^f^	Rat monoclonal IgG2b	1:50	Anti Rat IgG	1:500	ECs/Fb	[36, 38]
CD31	Surface	CO.3E-1D4^g^	Mouse monoclonal IgG2a-APC^c^	1:50	–	–	ECs	[23, 35]

^a^ECs: Endothelial cells / Fb: Fibroblasts

^b^CD34: marker for hematopoietic stem cells [35]

^c^CD31 conjugated to Allophycocyanin (APC) (direct staining)

^d^Supplied by Sigma-Aldrich

^e^Supplied by Biorbyt

^f^Supplied by Bio-Rad

^g^Supplied by NovusBio

### Characterization of the lytic cycle of *Besnoitia besnoiti* tachyzoites in primary BAEC and bovine fibroblasts

#### Invasion assays

Invasion assays were carried out essentially as previously described in MARC-145 cells [11]. Confluent monolayers of BAECs or fibroblasts (10^5^ cells/well) were seeded on sterile coverslips in P24 cell culture plates and were infected with 10^3^ viable tachyzoites/well (multiplicity of infection, MOI = 0.01). Next, at 4, 8, 12 and 24 h post-infection (hpi), 3 washes with PBS were performed to remove non-adherent extracellular tachyzoites, and three infected wells were left undisturbed without washing for 72 h. Infected cultures were further incubated for 72 h at 37 °C with 5% CO_2_ in a humidified incubator. Then, the cells were fixed using ice-cold methanol for 20 min at RT, and immunostaining was performed. Afterwards, the number of invasion events per well, namely, small and large parasitophorous vacuoles (PVs) and lysis plaques (LPs), was counted as previously described [11]. The invasion of a single tachyzoite was assumed to result in one invasion event. All conditions were tested in triplicate in at least three independent experiments.

#### Proliferation assays

P24 cell culture plates with confluent BAECs or fibroblast monolayers (10^5^ cells/well) were infected with 10^6^ purified viable tachyzoites/well (MOI = 10) [11]. After 4 h, the wells were washed 3 times with PBS to remove non-adhered extracellular tachyzoites, and the infected culture plates were further incubated at 37 °C in a humidified 5% CO_2_ incubator. At 4, 8, 12, 24, 48 and 72 hpi, supernatants were discarded, and samples were collected according to the manufacturerʼs instructions from a Rapid Lyse Kit (Mackerey-Nagel, Düren, Germany) and stored at -80 °C until DNA extraction. In parallel, cell culture replicates were seeded on coverslips, infected as described above and fixed with a mixture of 3% paraformaldehyde and 0.05% glutaraldehyde in PBS at the pi times selected (4, 8, 12, 24, 32, 48 and 72 hpi) for immunofluorescence staining. Three coverslips were photographed for each condition using an inverted fluorescence microscope at 600× magnification (Nikon Eclipse TE 200, Chiyoda, TYO, JP). Proliferation assays were carried out in triplicate and repeated in three independent experiments.

#### *Besnoitia besnoiti* invasion and proliferation in BAEC infected with BVDV

To study the possible influence of BVDV on *B. besnoiti* tachyzoite *in vitro* behaviour, BAECs were infected with a non-cytophatic strain of BVDV using supernatants obtained from persistently infected CPAE cells (ATCC® CCL-209™) (BVDV RT PCR Cq values of 18–20) [25]. Briefly, 1 ml of the supernatant from confluent CPAE cells was added to a confluent T25 flask of 90% confluent BAECs, incubated for 1 h at 37 °C and 5% CO_2_ and replaced with fresh culture medium. Infection was confirmed by RT-PCR [25] after two consecutive passages and at the end of the study.

This cell line, named BVD-BAEC, was kept under the same conditions but was physically separated from BVD-free cells, using a different incubator and a flow hood that were located in a separate room.

Afterwards, the effect of BVDV co-infection on the early (4 hpi) and late invasion (24 and 72 hpi) of *B. besnoiti* tachyzoites was studied. PD10-purified tachyzoites of the BbSpain1 isolate were inoculated into confluent BVDV-infected BAECs at a parasite MOI of 0.01, and at 4 and 24 hpi, non-invaded tachyzoites were removed with three washes with PBS. Three wells were left undisturbed without washing, and plates were fixed at 72 hpi. Proliferation assays were performed essentially as described for the lytic cycle characterization, with a MOI of 10, and samples were collected for subsequent DNA extraction and qPCR at 8, 24 and 72 hpi. In parallel, cultures that had been seeded on sterile coverslips were infected under the same conditions and fixed at the post-infection times previously mentioned. All conditions were tested in triplicate in three independent assays.

### Immunofluorescence staining

For immunofluorescence staining, supernatants of the cell cultures were discarded at 72 hpi. Next, cells were washed 3 times with PBS for 5 min each and then fixed by the addition of ice-cold methanol for 10 min. After another wash with PBS, cells were permeabilized with 300 µl/well of 0.2% Triton-X 100 in PBS for 30 min at 37 °C, followed by 3 additional washes with PBS. Primary polyclonal rabbit-anti tachyzoite BbSpain1 polyclonal antiserum [28] was added at a dilution of 1:1000 in PBS and incubated for 1 h at 37 °C. After 3 additional washes with PBS, Alexa Fluor® 594 goat anti-rabbit IgG (H+L), (Life technologies, Thermo Fisher Scientific, USA) was added to each well at a dilution of 1:1000 in PBS. The plates were incubated for 45 min at RT in the dark and washed 3 times with PBS. During the final wash, nuclei were labelled with DAPI staining. Finally, the plates were washed with distilled water, and the total number of invasion events per well was counted using an inverted fluorescence microscope (Nikon eclipse TE200) at 200× magnification.

### DNA extraction and quantitative real-time PCR (qPCR)

The harvested cell culture samples were incubated for 10 min at 56 °C, and DNA was purified using the spin column protocol for cultured cells according to the manufacturerʼs instructions in the Rapid Lyse kit (Mackerey Nagel, Düren, Germany). DNA was eluted in 100 µl elution buffer. The DNA content and purity of each sample were measured by UV spectrometry using a Biotek Multiplate Reader (Biotek, Winooski, VT, USA).

A BbRT2 qPCR assay was carried out for the specific detection of *Besnoitia* spp. DNA from ungulates (i.e. *B. besnoiti*, *B. tarandi*, *B. caprae* and *B. bennetti*) and was performed as previously described [29]. Herein, the GoTaq (Promega, Madison, WI, USA) system was used. Briefly, each 25 μl reaction contained 12.5 μl of GoTaq master mix® (Promega, Madison, WI, USA), 0.5 μl of primer Bb3 (5′-CAA CAA GAG CAT CGC CTT C-3′; 20 μM), 0.5 μl of primer Bb 6 (5′-ATT AAC CAA TCC GTG ATA GCA G-3′; 20 μM), and 6.5 μl water. The qPCRs were run on a 7500 Fast Real-Time PCR System® (Applied Biosystems, Thermo Fisher Scientific, Waltham, MA, USA). Twenty-one hundred nanograms of DNA in a volume of 5 μl was added to each reaction.

The DNA positive control was extracted from *B. besnoiti* tachyzoites cultured *in vitro*. The product of the DNA extraction process using water instead of cells was used as a negative control. In each qPCR, 10-fold serial dilutions of genomic DNA corresponding to 0.1–100,000 Bb-Spain1 tachyzoites were included. The cycling conditions were 10 min at 95 °C, followed by 40 cycles at 95 °C for 15 s and at 60 °C for 1 min. Fluorescence emission was measured during the 60 °C step. A dissociation stage was added at the end of each run, and the melting curves were analysed. The threshold cycle values (Cq-values) obtained for positive samples in the BbRT2-PCR are also expressed as tachyzoites per reaction using the standard curve included in each run.

To normalize the quantification of the parasites and account for variations in the DNA content in the samples, a bovine ß-actin standard curve was designed ranging from 64 ng of DNA per μl to 0.2 ng per μl. The results are expressed as the ratio between the amount of parasites and bovine ß-actin.

### Data analyses

To assess the differences in parasite invasion and proliferation among the different time points pi studied for each cell line, Kruskal-Wallis followed by a Dunnʼs *post-hoc* test was performed. Additionally, differences among the cell lines were explored using a Mann-Whitney U-test.

The doubling time (Td) was defined as the period of time required for a tachyzoite to duplicate during the exponential multiplication period, excluding the lag phase (when there is no parasite multiplication) and the egression phase. The doubling time was determined by using non-linear regression analysis and an exponential growth equation, as previously described [30].

Chi-square test was used to address possible differences regarding the percentages of the different invasion and proliferation outcomes between BAECs and fibroblasts and among BVDV-infected BAECs and non-infected BAECs. Statistical analyses were performed with GraphPad statistics software 6.0 (San Diego, CA, USA).

## Results

### Results show the endothelial and fibroblast origins of the primary cell cultures

**Fig. 1:**
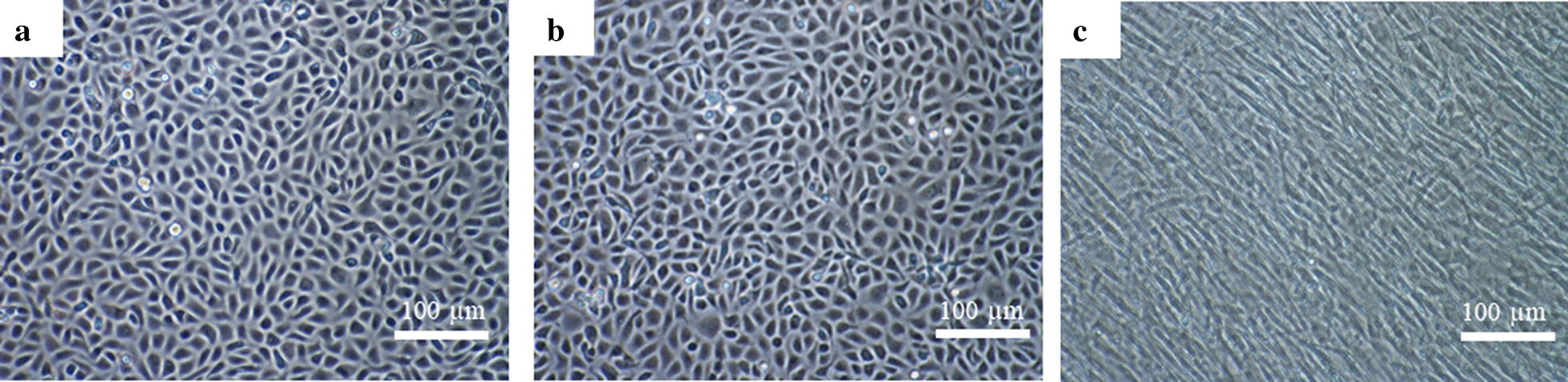
Confluent monolayers of primary low-passage BAEC (**a**); high-passage BAECs (**b**) and bovine fibroblasts (**c**) at 100× magnification under an inverted light microscope. *Scale-bars*: 100 µm

Recently isolated BAECs showed a polygonal morphology and grew in confluent monolayers with a cobble stone-like pattern. BAECs (Fig. 1a) were not contaminated with other cell types, such as fibroblasts or smooth muscle cells, which are frequent contaminants after the chemical digestion of the endothelia. BAECs were viable and successfully passaged and maintained *in vitro* up to passage 30, without remarkable morphological changes, as shown in Fig. 1b. Bovine fibroblasts presented an elongated morphology and grew in non-overlapping monolayers, as shown in Fig. 1c.

**Fig. 2:**
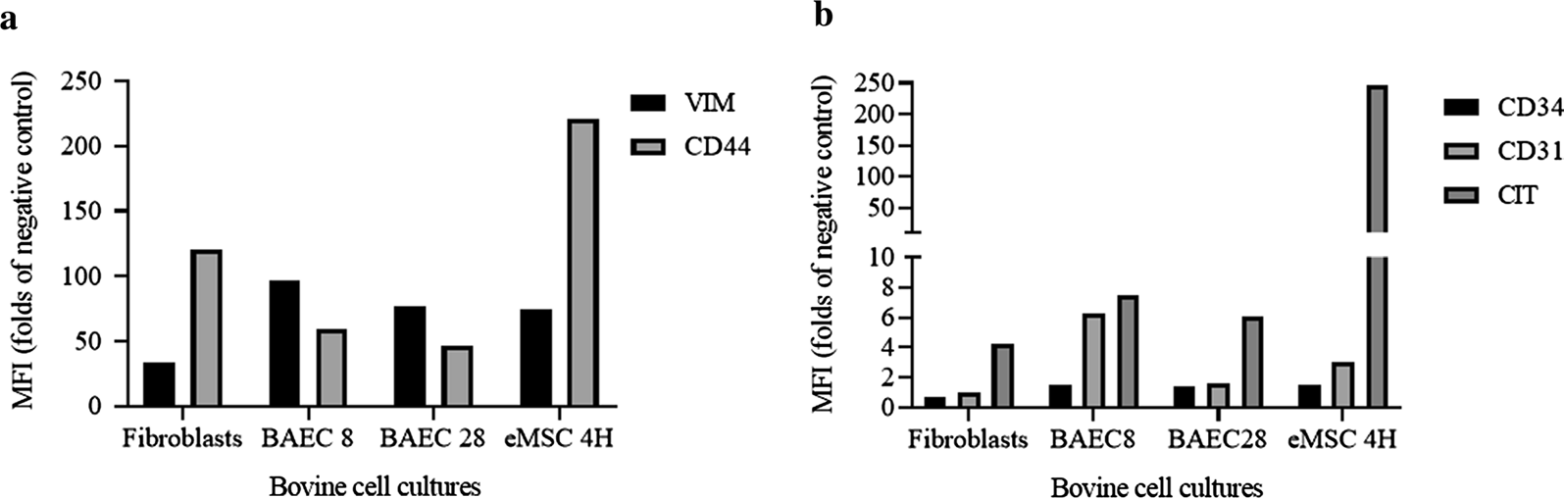
Intracellular and surface marker expression patterns in primary BAECs and fibroblasts by flow cytometry. **a** CD44 and Vimentin labelling. **b** CD31, CD34 and Cytokeratin labelling. Data correspond to the mean fluorescence intensity (fold-change compared to the negative control) for each sample

Flow cytometry analyses showed that low-passage BAECs were strongly positive for CD31, CD44, vimentin and cytokeratin, whereas they presented low expression of CD34 (Fig. 2). The expression pattern of cellular markers showed by high-passage BAECs was similar to that of low-passage BAECs for vimentin, CD44 and cytokeratin, but CD31 showed a lower fold-change in expression in high-passage BAECs (Fig. 2). Additionally, high-passage BAECs were found to be negative for CD34. Fibroblasts were positive for cytokeratin, vimentin and CD44 and negative for CD31 and CD34 (Fig. 2). The pattern of expression of intracellular and surface markers for the bovine endometrial eMSCs 4H consisted of the high expression of vimentin, cytokeratin and CD44, but these cells remained negative for CD34 and CD31 (Fig. 2).

### *Besnoitia besnoiti* displays higher invasion rates in BAECs at late invasion

**Fig. 3:**
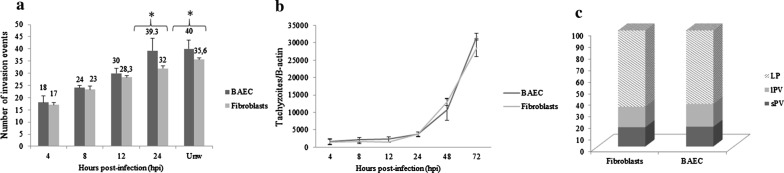
**a** *Besnoitia besnoiti* tachyzoite invasion rates in BAECs and bovine fibroblasts. The total number of invasion events (parasitophorous vacuoles and lysis plaques) per well at the different time points pi. **b** *In vitro* proliferation kinetics of *B. besnoiti* tachyzoites in BAECs and bovine fibroblasts, as determined by qPCR. **c** Invasion and proliferation outcomes (small and large parasitophorous vacuoles and lysis plaques) of *B. besnoiti* tachyzoites in BAECs and bovine fibroblasts in 24 hpi washed wells. *Abbreviations*: sPV, small parasitophorous vacuole; lPV, large parasitophorous vacuole; LP, lysis plaque

The total number of invasion events (Lysis plaques -LPs- and/or parasitophorous vacuoles -PVs) at 72 hours post-infection (hpi) ranged from 18 in wells that were washed at 4 hpi to 39 in wells that were washed at 24 hpi in BAECs and from 17 in wells that were washed at 4 hpi to 32 in wells that were washed at 24 hpi in fibroblasts. A significant increase in the number of events per well was shown in both cell cultures when the invasion times that were assayed were compared up to 24 hpi (Kruskal-Wallis H-test followed by Dunnʼs multiple comparison test, *χ*^2^ = 40.73, *df* = 4, *P* < 0.001 in BAECs and *χ*^2^ = 41.14, *df* = 4, *P* < 0.001 in fibroblasts) (Fig. 3a). However, statistically significant differences between wells that were washed at 24 hpi and unwashed wells (at 72 hpi) were not observed (Mann-Whitney U-test, *U*_(9)_ = 21.50, *Z =* 1.351, *P* = 0.997 in BAECs and *U*_(9)_ = 20, *Z =* 1.329, *P* = 0.0716 in fibroblasts).

When infected BAECs and fibroblasts were compared, statistically significant higher invasion rates were found in BAECs at 24 hpi (Mann-Whitney U-test, *U*_(9)_ = 10, *Z =* 2.646, *P* = 0.005) and 72 hpi (in unwashed wells) (Mann-Whitney U-test, *U*_(9)_ = 8, *Z =* 2.669, *P* = 0.0021) (Fig. 3a). The invasion rates were similar at 4, 8 and 12 hpi. Moreover, up to 50% of invasion events in both cell lines occurred between 4 and 8 hpi, as shown in Fig. 3a.

Tachyzoites from the Bb-Spain 1 isolate showed an exponential growth in both primary cell cultures assayed (*R*^2^ > 0.95) (Fig. 3b) from 12 hpi onwards, with a significant increase in the number of tachyzoites per well from 24 hpi onwards in both BAECs (Kruskal-Wallis test followed by Dunnʼs multiple comparison test, *χ*^2^ = 46.12, *df* = 5, *P* < 0.0001) and fibroblasts (Kruskal-Wallis test followed by Dunnʼs multiple comparison test, *χ*^2^ = 45.83, *df* = 5, *P* < 0.0001). The doubling time during the exponential growth phase of the tachyzoites of the Bb-Spain-1 isolate was 13.15 ± 2.34 h in BAECs and 13.34 ± 2.17 h in bovine fibroblasts. No significant differences were found among BAECs and fibroblasts when tachyzoite yields (tachyzoites/β-actin) for each post-infection time point were compared (Mann-Whitney U-test: *U*_(9)_ = 34, *Z =* 0.3010, *P* = 0.6048 at 4 hpi; *U*_(9)_ = 23, *Z =* 0.9256, *P* = 0.1359 at 8 hpi; *U*_(9)_ = 18, *Z =* 0.7977, *P* = 0.503 at 12 hpi; *U*_(9)_ = 27, *Z =* 0.2182, *P* = 0.2581 at 24 hpi; *U*_(9)_ = 35, *Z =* 0.08278, *P* = 0.665 at 48 hpi; and *U*_(9)_ = 22, *Z =* 0.2784, *P* = 0.1081 at 72 hpi).

**Fig. 4:**
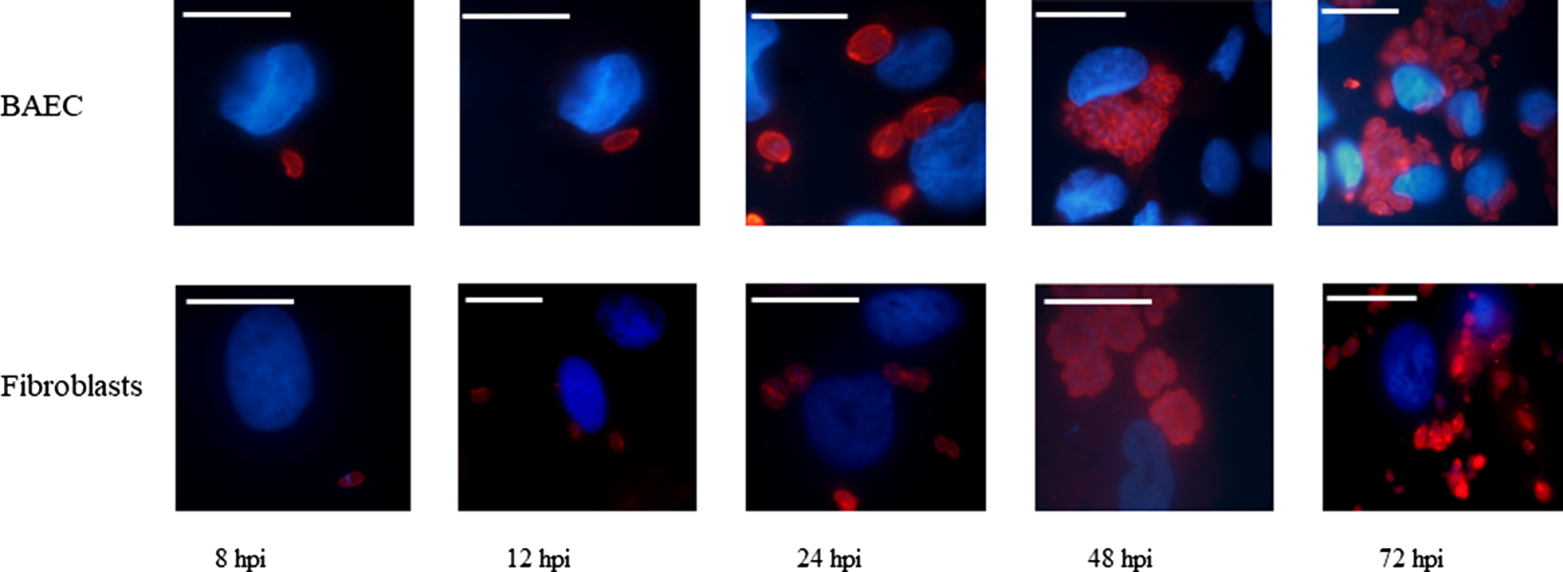
Lytic cycle of *Besnoitia besnoiti* tachyzoites, as monitored by immunofluorescence in primary BAECs and fibroblasts from 4 hpi up to 72 hpi. *Besnoitia besnoiti* tachyzoites are stained with a rabbit polyclonal antibody against *B. besnoiti* (red), and the nuclei of host cells are stained with DAPI. *Scale-bars*: 20 µm

The results from qPCR were in agreement with the immunostaining images captured at the same time points pi (Fig. 4). Tachyzoites had already invaded host cells between 4 and 8 hpi, followed by a lag phase of up to 24 hpi. Then, proliferation began inside PVs that initially contained 2 tachyzoites (Fig. 4), and at least two rounds of replication had been completed by 32 hpi. PVs continued to grow, and large PVs, as well as parasite egress and LPs, were observed from 48 hpi onwards in both BAECs and fibroblasts.

The parasite invasion and proliferation outcomes that were analysed at 72 hpi in 24 h washed plates mostly consisted of LP (*c.*65%, Fig. 3c), rather than PVs, without statistically significant differences between the cell lines (Chi-square test, *χ*^2^ = 0,1676, *df* = 2, *P* = 0.9196).

### BVDV co-infection facilitates the early invasion of *B. besnoiti* tachyzoites in BAECs

First, the BVDV-infection status was checked, showing BVDV RT-PCR Cq values ranging from 18 to 20 for BVDV-BAECs at every time point checked, while BAECs remained negative (Cq values over 38) (data not shown).

**Fig. 5:**
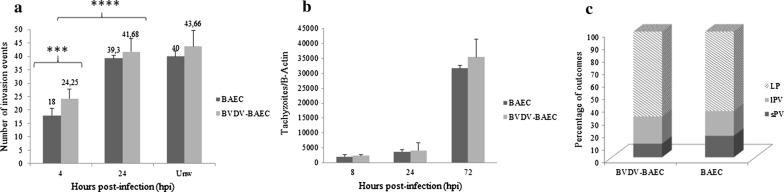
**a** *Besnoitia besnoiti* tachyzoite invasion rates in BVDV-BAECs and BAECs. The total number of invasion events (large and small parasitophorous vacuoles and lysis plaques) per well at the different time points pi is shown. **b** *In vitro* tachyzoite yields of *B. besnoiti* in BVDV-BAECs and BAECs, as determined by qPCR. **c** Invasion and proliferation outcomes (small and large parasitophorous vacuoles and lysis plaques) of *B. besnoiti* tachyzoites in BVDV-BAECs and BAECs in 24 hpi washed wells. *Abbreviations*: sPV, small parasitophorous vacuole; lPV, large parasitophorous vacuole; LP, lysis plaques

The total number of invasion events (LPs and/or PVs) at 72 hpi ranged from 26 in wells that were washed at 4 hpi to 48 in wells that were washed at 24 hpi in BVDV-BAECs. A significant increase in the number of invasion events was observed at 24 hpi compared to 4 hpi (Mann-Whitney U-test, *U*_(9)_ = 0, *Z =* 3.049, *P* < 0.0001) (Fig. 5a). The maximum number of invasion events was found in unwashed wells, although there were no significant differences compared with that of wells that were washed at 24 hpi (Mann-Whitney U-test, *U*_(9)_ = 21.50, *Z =* 1.131, *P* = 0.0962). At 4 hpi, more than 50% of the invasion outcomes had already been produced (Fig. 5a).

When BVDV-infected BAECs and BAECs were compared, statistically significant higher invasion rates were found in BVDV-infected BAECs than in BAECs in wells that were washed at 4 hpi (Mann-Whitney U-test, *U*_(9)_ = 0, *Z =* 2.409, *P* < 0.001) (Fig. 5a).

The tachyzoite yield was similar between BVDV-infected BAECs and BAECs at 8 and 24 hpi. However, the tachyzoite yield was higher in BVDV-BAECs than in BAECs at 72 hpi, although the differences were not statistically significant (Fig. 5b; Mann-Whitney U-test, *U*_(9)_ = 27, *Z =* 0.4045, *P* = 0.2581).

The parasite invasion and proliferation outcomes mostly consisted of LP (*c.*65%, Fig. 5c), rather than PVs at all times assayed in both BAECs and BVD-infected BAECs, without statistically significant differences between the cell systems (Chi-square test, *χ*^2^ = 2.03, *df* = 2, *P* = 0.3616).

## Discussion

Herein, two primary bovine cell lines, BAECs and fibroblasts, that were free from frequent cell culture contaminants were obtained and characterized by flow cytometry for the first time to study *B. besnoiti* infection in target cells.

The development of *in vitro* models for the study of obligate intracellular protozoans that utilize target primary cells is timely and essential [7], since immortalized cell lines show phenotypic modifications that are characteristic of tumour-like cells and, consequently, may lose the phenotypic traits and specific functions of their tissue of origin [12]. In addition, the host species origin and tissue localization are critical to better model *in vivo* environments. Previous studies carried out with ECs and fibroblasts infected with *B. besnoiti* have been restricted to either BUVECs [13, 14] or HFF cells [11]. However, both cell lines do not fulfil both the host species and tissue location requirements. Finally, another crucial requirement that was considered was the absence of widespread BVDV. Thus, herein, an exhaustive two-step quality control was performed. First, the health status of donors, which is usually unknown for primary and established bovine cell lines that are available in different repositories worldwide, was carefully checked [18]. Next, the foetal calf serum was shown to be free of BVDV, since it is widely known that non-cytopathic strains are usually present in the foetal bovine serum batches that are available worldwide [31].

When both cell lines were characterized, the morphology results and cytometry markers confirmed the endothelial and fibroblast origin. Endothelial cells from all vascular beds are aligned longitudinally *in vivo*, forming a single layer due to their anatomical location and laminar flow. However, this morphology may change as a result of *in vitro* conditions and is not considered a reliable indicator [32]. BAECs grew as a single layer of cells and presented a cobblestone pattern at confluence, as described for ECs from large vessels [32]. In contrast, fibroblasts presented a flattened morphology and were spindle-shaped, as expected [33]. Moreover, each cell line showed a distinct expression pattern of intracellular and surface markers, and in agreement with previous reports, CD31 (or PECAM1) was the surface marker that best discriminated between BAECs and fibroblasts since fibroblasts lacked the expression of CD31 [23]. Additionally, differences in CD34 labelling were detected, and only low-passage BAECs were slightly positive. The CD31 and CD34 labelling of different ECs has been reported. CD31 is the most classical endothelial marker [23] and is expressed at cell junctions to maintain endothelial integrity and control permeability [34]. In contrast, CD34 is a marker of pluripotency that is found only in circulating endothelial precursors [35]. Interestingly, the CD31 and CD34 labelling also differed between low-passage and high-passage BAECs despite the homogeneous morphology shown by both cell lines at various passages. The lack of CD34 labelling may have been influenced by *in vitro* selection after 28 passages. This finding strengthens the hypothesis that the use of low-passage primary cells can avoid the phenotypic changes associated with *in vitro* selection. In agreement, the use of commercial primary ECs with a passage number higher than 16 is not recommended. In contrast, CD44, vimentin and cytokeratin labelling were observed regardless of the cell line origin and passage number. CD44 is a ubiquitous ligand that is important for the regulation of endothelial cell proliferation and apoptosis, modulating CD31 and VE-cadherin expression [36], and for the regulation of vascular endothelial integrity *via* a CD31-dependent mechanism [37]. In fibroblasts, CD44 mediates proliferation, apoptosis and migration [38]. Vimentin is an intermediate filament that provides mechanical integrity and structural support to fully differentiated endothelial cells [39]. It has also been shown that vimentin is essential for the differentiation of endothelial cells from embryonic stem cells [40]. The network of cytoskeletal proteins is essential for the response of endothelial cells to mechanical stimuli, and the remodelling of this network may be important for the adhesion of motile tachyzoites to the endothelium, as has been shown for *T. gondii* tachyzoites under shear forces *in vitro*, where adhesion was enhanced [41]. In fibroblasts, vimentin is a key regulator of cell proliferation and keratinocyte differentiation in wound healing [42]. Finally, cytokeratin is a marker that is commonly associated with epithelial cells, but early studies performed in endothelial cells from bovine aortas described a mixed population, composed of cells that were positive and negative for cytokeratin [43]. Cytokeratin-positive cells were more polygonal and grew in a cobblestone pattern that is commonly associated with endothelial cells, as observed in this study. In addition, the different phenotypes of endothelial cells have been described according to the cytokeratin pattern, and cytokeratin-positive cells in the microvasculature have been thought to be danger-sensing cells [44]. In fibroblasts, cytokeratin expression is reported to be low, but some keratins have been shown to be present in fibroblasts *in vitro* [45].

Regarding the *in vitro* parasite behaviour, tachyzoites were able to successfully invade and replicate within both of the target bovine cell lines that were assayed, as expected. Moreover, the parasites showed almost an identical lytic cycle to that previously described in MARC-145 cells [11, 46]. Generally, our findings suggest that neither invasion nor proliferation are particularly favoured in both bovine target cells compared to a non-bovine cell line. According to these results and previous studies carried out with tachyzoites of the BbSpain-1 isolate, tachyzoites display lower invasion and proliferation rates than both *N. caninum* [47] and *T. gondii* [48] in all cell types that have been assayed thus far. Thus, this isolate was categorized as a low-invasion and low-proliferation isolate. However, slight differences between the invasive capabilities in BAECs and fibroblasts were found since the invasion rates at 24 hpi and in unwashed wells were higher in BAECs than in fibroblasts. Accordingly, the maximum IR that was found in the present work was observed in BAECs and was not higher than 4.5%, in contrast with observations previously made [14], which showed IRs over 30% in BUVECs that were infected with tachyzoites from the Bb1Evora04 isolate. This result may have been influenced by several factors, such as differences in the isolate, multiplicity of infection (MOI) and passage number used. Moreover, higher MOIs were used, and the number of *in vitro* passages was not specified for the Evora04 isolate of *B. besnoiti* [14]. In a previous study, differences in IRs were observed among different isolates, in which a Portuguese isolate showed higher IRs (approximately 20%) than the Spain1 isolate in MARC-145 cells [11]. It has been reported that prolonged *in vitro* culture can alter the phenotypic features and even the virulence of isolates of *N. caninum* [49] and *T. gondii* [50]. Our results also showed that the tachyzoite invasion of the host cell can take place up to 24 hpi since there was an increase in the number of invasion events up to 24 hpi, confirming the prolonged extracellular survival of tachyzoites. However, most invasion events took place before 24 hpi, showing that the invasion kinetics that were observed in both bovine target cell lines were similar to those in MARC-145 cells, where most of the invasion outcomes occurred between 4 and 8 hpi. Again, exponential growth was observed, and replication was initiated from 24 hpi onwards, so the lag phase that is required for the parasite to adapt to the intracellular conditions extended up to 24 hpi. In addition, Td was in agreement with previous studies [11, 46]. The presence of both PVs and LPs at 72 hpi suggests asynchronous growth, which is most likely associated with the prolonged invasion capabilities of tachyzoites [11].

However, the *in vitro* studies of the closely related apicomplexan parasites have shown host-type specific *in vitro* behaviour. The tachyzoite to bradyzoite switch seems to be favoured in differentiated cell types, such as keratinocytes for *N. caninum* [51] or skeletal muscle cells in the case of *T. gondii* [52]. Additionally, in *Eimeria* spp., the formation of meronts I has only been described in cells with a bovine origin, both for epithelial and endothelial cells [53]. In the cattle pathogen *N. caninum*, no striking differences were found when the behaviour of tachyzoites from high virulence (NcSpain7) and low virulence (NcSpain1H) isolates was studied in cells with a different host origin (MARC-145 *vs* bovine trophoblast). However, differences were found among two bovine cell lines from the placenta, foetal trophoblasts and maternal caruncular cells, since higher IRs and tachyzoite yields were found in trophoblast cells, suggesting that there was a barrier function for caruncular cells [30]. Considering the heterogeneity of both ECs and fibroblast populations [17, 54], further refinement of the *in vitro* models that were standardized herein could be possible, including cell lines from target locations where parasite growth is favoured *in vivo*, such as the microvasculature from nasal turbinates, testis or skin.

Finally, to our knowledge, this is the first study in which the *in vitro* interaction of BVDV with a protozoan belonging to the subfamily Toxoplasmatinae has been studied. Interactions between *B. besnoiti* and BVDV could be relevant in the pathogenesis of bovine besnoitiosis, with consequences at the molecular level and in the clinical presentation of the disease under field conditions. In several regions of Spain, BVDV is frequently circulating in herds [55], and its immunosuppressive properties are widely known since this virus is capable of altering both innate and adaptive responses in cattle [56, 57]. However, the possible synergism of *B. besnoiti* and BVDV coinfections is unknown.

In the present study, despite a slight difference in the early tachyzoite invasion of BAECs, there was no difference in tachyzoite yields observed in BVDV-BAECs compared to BAECs. Additionally, the total number of invasion events remained unchanged. These results may have been due to the immunosuppressive properties of this virus that have been described *in vitro* so far, mostly affecting the early recognition of pathogens that are related to TLR signalling; additionally, a decrease in Myeloid Differentiation factor 88 (MyD88) expression has been observed, which resulted in the impaired leukocyte function of myeloid cells [58]. Interestingly, MyD88 has been shown to confer resistance in mice against closely related Toxoplasmatinae parasites such as *T. gondii* [59] and *N. caninum* [60].

Although BVDV infection has been suggested to be a risk factor that may facilitate or exacerbate other infectious diseases, studies performed *in vivo* thus far have provided variable results. Several studies that have compared coinfections with BVDV and other closely related parasites, such as *N. caninum*, have found an association with BVDV and abortions due to *N. caninum* [61, 62], while other studies claimed that there was no association [63]. Regarding *in vitro* co-infections, a recent study performed with the intracellular pathogen *Mycoplasma bovis* demonstrated that BVDV infection did not influence the *in vitro* behaviour of the bacteria, as assessed in bovine macrophages (Bomac) [18]. Thus, epidemiological studies addressing coinfections with *B. besnoiti* and BVDV are necessary to demonstrate the possible synergism that has been suggested *in vitro*. Also, studying the interactions between *B. besnoiti* and BVDV is timely and could shed light on pathogenesis studies in other relevant bovine pathogens. One of the most relevant pathogens that is characterized by endothelial damage is the Blue Tongue Virus, and numerous studies have been performed in bovine pulmonary artery endothelial cells without checking for the absence of pestivirus infection [64].

In summary, we have standardized two *in vitro* models that employ the primary target bovine cells of acute and chronic *B. besnoiti* infection. The detailed description of the parasite lytic cycle in both cell lines could be used as the basis for further host-pathogen interaction studies at the molecular level. Moreover, the BVDV-BAEC model could be useful for other Toxoplasmatinae parasites and for other ruminant pathogens that replicate in ECs.

## Conclusions

In the present work, we have characterized two novel standardized *in vitro* models for *Besnoitia besnoiti* infection based on recently isolated bovine primary target BAECs and fibroblasts. The relevance of BVDV coinfections has been evidenced and should be considered in further studies with other cattle pathogens.

## Data Availability

Data supporting the conclusions of this article are included within the article.

## Abbreviations

APC: allophycocyanin
BAEC: bovine aortic endothelial cells
Bomac: bovine macrophages
BUVEC: bovine umbilical vein endothelial cells
IR: invasion rate
qPCR: quantitative PCR
BVDV: bovine viral diarrhea virus
CPAE: calf pulmonary artery endothelial cells
Cq: quantification cycleDAPI: 4’,6-diamidino-2-phenylindole
DMEM: Dulbeccoʼs modified Eagleʼs medium
hpi: hours post-infection
DNA: deoxyribonucleic acid
ECs: endothelial cells
EDTA: ethylenediaminetetraacetic acid
ELISA: enzyme-linked immunosorbent assay
FBS: fetal bovine serum
IFAT: immunofluorescent antibody test
KH-R: embryo bovine heart
LP: lysis plaque
LVES: large vessel endothelial supplement
MOI: multiplicity of infection
PBS: phosphate-buffered saline
PV: parasitophorous vacuole
RPMI: Roswell Park Memorial Institute medium
RT: room temperature
RT-PCR: retrotranscriptase PCR
TNB: Tris-NaCl-blocking buffer
UV: ultraviolet
